# Artificial intelligence and neurodegeneration: state of the art and explainability

**DOI:** 10.1186/s40942-026-00807-4

**Published:** 2026-05-19

**Authors:** Emanuele Crincoli, Maria Cristina Savastano, Alfonso Savastano, Matteo Mario Carlà, Federico Giannuzzi, Fiammetta Catania, Raphael Kilian, Clara Rizzo, Stanislao Rizzo

**Affiliations:** 1 Ophthalmology Unit, “Fondazione Policlinico Universitario A. Gemelli IRCCS”, Catholic University “Sacro Cuore”, Largo A. Gemelli 8 - 00198, Rome, Italy; 2 Departement d’Ophtalmologie, Hopital Fondation Adolphe De Rothschild, 29 Rue Manin, Paris, 75019 France; 3 Departement of Translational Medicine, University of Ferrara, Ferrara, Italy; 4 Ophthalmic Unit, Department of Neurosciences, Biomedicine and Movement Sciences, University of Verona, 37219 Verona, Italy

**Keywords:** Artificial intelligence, Oculomics, Neurodegeneration, Alzheimer, Parkinson, Retina, Optical coherence tomography, Color fundus photograph, Deep learning, Explainability

## Abstract

**Background:**

Alzheimer disease (AD) and Parkinson disease (PD) are an increasing healthcare concern and growing cause of disability in our century. The advent of the artificial intelligence (AI) era and the early changes in retina and optic nerve morphology detected in neurodegenerative diseases by recent literature opened the way to the perspective of a AI-based diagnosis for both conditions.

**Main body:**

In AD a generalized decrease in macular Ganglion Cells-Inner Plexiform Layer (GC-IPL) and retinal nerve fiber layer (RNFL) and peripapillary RNFL thickness has been documented. Moreover, clinical AD is characterized by a lower choroidal thickness and higher choroidal vascularity index. AI can be predicted with around 80% accuracy based on small fundus vessels characteristics. Similarly, AI based on Optical Coherence Tomography (OCT) images showed very good performance in detecting AD, especially based on total macular volume (TVM) and GCL thickness. A combination of OCT and color fundus photographs (CFP) imaging reached 85% accuracy in detecting MCI. In PD typical retinal anomalies include a generalized thinning of the peripapillary RNFL with a degeneration pattern similar to the one of mitochondrial optic neuropathies, foveal pit widening, generalized retinal and retinal pigmented epithelium thinning and outer plexiform layer thickening. AI CFP-based detection of PD showed only 65% accuracy due to a low specificity, while multimodal imaging-based AI was the best approach, reaching a 100% sensitivity and 85% specificity.

**Conclusion:**

In consideration of the overall very good performance, AD and PD are likely to benefit from the support of retinal-imaging based AI for early diagnosis. To optimize the performance, especially in terms of specificity, the use of multimodal imaging and possible integration with meta-data has been demonstrated to be the best technique, especially for PD diagnosis. However, training and testing on a larger sample size and longitudinal evaluation of the chosen models will be needed before application on general population. So far, explainability of the proposed models seems robust and coherent with the corresponding central nervous system damage.

## Introduction

Neurodegenerative diseases are an increasing healthcare concern and a growing cause of disability in our century. Alzheimer disease (AD) and Parkinson disease (PD) are respectively the first and second most prevalent neurodegenerative diseases worldwide [1]. They are responsible for a significant human and socio-economic burden [2, 3] and their prevalence is expected to double by 2050 in both industrialized [4, 5] and non-industrialized countries [6, 7]. Around 15 million people are expected to suffer from mild cognitive impairment (MCI) due to AD or clinical AD by 2060 in the United States alone [5]. The advent of the era of artificial intelligence (AI) and the early retina and optic nerve changes described in recent literature have opened the possibility of AI-based diagnostic approaches for both conditions. Such approaches not only may reduce the costs associated with AD and PD, but could also enable unprecedented early diagnosis. In fact, on one hand, there is currently no validated, cost-effective and sufficiently accurate screening method for AD and PD, and on the other hand, ocular fundus analysis performed by the human eye is not accurate enough to ensure adequate screening standards.

A 2022 survey highlighted that the presence of a family history of the disease, the perceived risk associated with the condition, and most importantly, the perceived benefits of screening, are the main factors influencing potential adherence to AI-based screening protocols for neurodegenerative diseases [8]. In this perspective, PD is an excellent candidate for AI-based screening, given the beneficial impact of early diagnosis and treatment, the perceived disability associated with the disease and the frequent presence of a family history in affected individuals [9]. As concerns AD, the advent of monoclonal antibody treatment has renewed hope in slowing disease progression, making early diagnosis particularly valuable [10]. 

However promising, AI-based diagnosis and screening of PD and AD are still not part of routine clinical practice for several reasons. Among them are the need for substantial logistical changes within healthcare systems and the absence of consensus on the preferred and most effective screening strategy. Lastly, the intrinsic black box effect of deep learning (DL) technologies raises concerns about the trustworthiness of this approach. The aim of this review is therefore to illustrate the state of the art models proposed for automated diagnosis of both diseases and to provide elements of explainability of an AI-based diagnosis of AD and PD.

## Alzheimer disease

### Retinal changes in AD

Alzheimer disease is characterized by abnormal extracellular deposition of misfolded amyloid-β protein as amyloid plaques and intracellular accumulation of hyperphosphorylated tau proteins as neurofibrillary tangles within neuronal cells. The accumulation of these abnormal proteins leads to neurovascular unit impairment, oxidative stress and inflammation, ultimately resulting in microvascular damage, apoptosis and loss of neural tissue [11]. Such a degenerative process have been documented not only in the central nervous system but also in retinal neurons, especially in M-type retinal ganglion cells (RGCs). As a result, AD patients experience decreased contrast sensitivity, altered color and motion perception and homonymous visual field defects, which may sometimes represent the presenting symptoms of the disease (visual variant of AD) [12]. Neural loss in the retina is reflected by a generalized decrease in peripapillary retinal nerve fiber layer (ppRNFL) and macular RNFL thickness, as well as reduced macular volume [13]. Moreover, in clinical AD, Mini-Mental State Examination scores correlate well with TMV [14]. However, Ganglion Cells-Inner Plexiform Layer (GC-IPL) thickness has been shown to be even more sensitive for MCI and AD diagnosis than RNFL thickness [15]. In addition, the spatial distribution of GC-IPL loss appears to differ between MCI and AD patients, which may provide insight into disease progression [16]. 

Changes in retinal and choroidal vasculature have also been documented in association with AD. This is consistent with observations in the Central Nervous System, where the onset of dementia is often preceded by reduced cerebral blood flow [17]. Patients with clinical AD are characterized by reduced choroidal thickness [18] and a higher choroidal vascularity index.

### Results of AI in the diagnosis of AD

Several authors have assessed the feasibility of AD diagnosis based on AI applied to retinal imaging. A large part of the studies focused on the predictive potential of vascular features and therefore applied vessel segmentation. Tian et al. [19] investigated the possibility of diagnosing AD based on fundus vessels segmentation of UK biobank CFPs. The authors adopted a multi-step approach consisting of three phases (image quality selection, vessel segmentation and classification) using a Support Vector Machine (SVM) model. This design aimed to increase control over the automated process and improve explainability. Another strength of this study was its reliance on the high-quality dementia labeling in the UK biobank, which is based on a comprehensive evaluation rather than a single diagnostic test. Moreover, testing was performed using five different healthy control groups to increase the reliability of the results. Interestingly, while initial performance reached 68.2% accuracy, refinement with feature selection based on occlusion testing increased accuracy to 82.4%. This suggests that not all vascular regions are equally affected by disease progression; in fact, according to saliency maps, small vessels and capillary carry the most informative signal. Similarly, Zhang et al. [20] reported that vascular features extracted from fundus images assist in diagnosing mild cognitive impairment (MCI), a precursor of various forms of dementia including AD and PD. Other authors used the NFN+ deep learning framework for retinal vessel segmentation and measurement in patients with MCI. A nonlinear Random forest model was then applied to distinguish MCI subjects from the normal population based on vessel segmentation combined with additional variables. The overall performance of the model achieved an AUC of 0.799, with age, body mass index (BMI), global vein width, retinal vessel fractal dimension (FD), and central retinal artery equivalent (CRAE) ranked as the most important features [21]. Non-vessel-based approaches have also been developed. A CFP-based deep learning algorithm using an EfficientNet-b2 network was recently developed by Cheung et al. [22] using 12 949 images from 648 patients with AD and 3240 controls. In the testing datasets, the bilateral DL model achieved accuracies ranging from 79.6% to 92.1%. In datasets containing PET information, the model also distinguished amyloid β positive from amyloid β negative participants, with accuracies ranging from 80.6% to 89.3%.

Another approach involved models based on optical coherence tomography (OCT) segmentation. Wang et al. [23] used an XGboost model to identify AD patients based on the thickness of various retinal layers on OCT B-scan imaging. They demonstrated significant decrease in average, superior, and inferior quadrant ppRNFL, macular RNFL, ganglion cell layer (GCL) and inner plexiform layer (IPL) thicknesses, as well as TMV. Moreover, TMV was positively associated with Mini-Mental State Examination and Montreal Cognitive Assessment scores; IPL thickness was negatively correlated with medial temporal lobe atrophy; and GCL thickness was positively correlated with CSF Aβ_42_ /Aβ_40_ and negatively with p-tau level. Model accuracy reached 74% and TVM was identified as the most informative feature.

Nunes et al. [24] used an innovative approach based on texture features extracted from enface segmentations of each retinal layer to distinguish healthy subjects from individuals with PD or AD. Interestingly, they showed that texture analysis conveys information not captured by layer thickness alone. They also reported that, in AD, the superior macular region exhibits the most relevant textural alterations, while PD shows characteristic changes in the overall macular texture.

A combination of OCT and fundus imaging was used by Gao et al. [25] in a dual-stream attention neural network trained to distinguish healthy individuals from patients with MCI and AD. The algorithm reached 85% accuracy in detecting MCI.

In a recent study from Wisely et al. [26], a multimodal imaging approach was adopted for automated AD diagnosis. The included inputs were UWF color and autofluorescence imaging, OCT angiography (OCTA) of the superficial capillary plexus (SCP) and GC-IPL maps. Notably, the multimodal model performed similarly to the GC-IPL-only model (the best single input configuration), with an AUC of approximately 0.83.

## Parkinson disease

### Retinal changes in PD

Several retinal structures have been shown to undergo significant changes in patients developing PD, and these alterations become more pronounced in advanced stages of the disease [27]. As concerns the papilla, a generalized thinning of the ppRNFL and a shallower optic cup have been documented in PD eyes compared with healthy controls [27]. Unlike AD, in PD smaller RGCs and axons are preferentially affected, which results in a degeneration pattern similar to that observed in mitochondrial optic neuropathies [28]. The macroscopic correlate of this type of damage is the development of temporal pallor of the optic disc and a central or paracentral scotoma in the later stages of the disease [29]. The fovea also shows relevant abnormalities, including foveal pit widening, generalized retinal and retinal pigmented epithelium (RPE) thinning and outer plexiform layer (OPL) thickening. Foveal thinning primarily affects the inner layers and it is directly correlated with enlargement of the foveal avascular zone, motor impairment and disease duration [30]. The pattern of retinal degeneration is generally coherent with that detected in the central nervous system, with predominant damage in the hemiretina corresponding to the most affected hemisphere [27]. As a consequence of dopaminergic retinal neurons degeneration, PD patients frequently exhibit reduced contrast sensitivity and dyschromatopsia [31]. 

### Results of AI in the diagnosis of PD

Recently, several FL and DL models were trained and tested using CFPs from the UK biobank to perform automatic diagnosis of incident (consistent with pre-symptomatic and/or prodromal) PD and prevalent PD [32]. DL outperformed FL, with the AlexNet model showing the best classification results among all tested algorithms. However, overall performance was only moderately satisfactory: prevalent PD was detected with an accuracy of 65% and incident PD with an accuracy of 60%. In both categories, sensitivity was much higher than specificity (respectively 74.0% and 56.0% in prevalent PD and 64.0% and 56.0% in incident PD), suggesting a high rate of false positive cases. The cumulative model (prevalent and incident PD) based on AlexNet architecture reached an AUC of 0.77 (95% CI 0.74–0.81). Qualitative explainability analysis was based on the overlap of a backpropagation map with a segmentation map of the optic disc, macula and retinal vessels generated using Automorph. Explanation infidelity (INFD) and explanation sensitivity (SENS) were used as quantitative metrics to assess model reliability. Both methods revealed good explainability for the model based on AlexNet architecture, whereas GoogleNet, Inception-V3, and ResNet-50 were largely influenced by data perturbations. Nevertheless, the classification performance remained only partly satisfactory, which could be due either to the relatively small sample size (77 images per group for prevalent disease and 46 images per group for incident disease detection) or the use of CFPs as the sole source of information.

Richardson et al. [33] recently reported a much higher AUC of 0.918 using a single-eye model (with comparable results for right and left eyes) based on multimodal imaging, including optical coherence tomography (OCT) ganglion cell-inner plexiform layer (GC-IPL) thickness color maps, OCT angiography 6 × 6-mm en face macular images of the superficial capillary plexus (SCP), and ultra-widefield (UWF) fundus color and autofluorescence photographs. Once again sensitivity was higher than specificity (100% and 85% respectively). The size of the PD population was similar to that of Tran et al. [32] but the healthy control group was five times larger, and groups asymmetry was balanced using two different strategies (a modified binary cross entropy loss function and a weighted random sampler). Interestingly, UWF color photographs were the most effective imaging input, and GC-IPL thickness maps were the least contributory. However, the results of the UWF-only model were only slightly superior to those obtained using images from the UK biobank, suggesting that multimodal imaging was the key factor underlying the improved performance reported by Richardson et al. [33]

Diaz et al. [34] also tested the UK biobank dataset for automatic PD diagnosis and reported an accuracy of around 70% using a sigmoid SVM algorithm. They noted that saliency maps of the best-performing algorithms highlighted smaller caliber vessels as important regions for classification. Performance of machine learning algorithms based solely on OCT images has also been assessed. A random forest model based on overall macular thickness and retinal layer thickness achieved a 64% accuracy (external validation) in distinguishing healthy controls from PD [35]. However, performance improved substantially when using a texture based approach on enface images of different retinal layers, as described in previous paragraphs [24]. 

Despite these encouraging results, considering the blackbox effect, it is essential to ensure that retinal abnormalities highlighted by visualization methods correspond to true neurological damage. For this reason, neurological evaluation of patients classified as PD may reinforce trustworthiness of AI-based predictions. Evidence gathered so far is reassuring. Some authors [36] demonstrated the feasibility of performing automated assessment of neurologic dysfunction in PD patients using a combination of general information (age, sex, comorbidities) and CFPs. In that study, accuracy in predicting the Hoehn and Yahr scale score was 70.45% in the external validation setting, with the best performance obtained using ResNet-18 architecture.

Lastly, a novel approach was reported by Wolf et al. [37], who used aqueous humor proteomics to detect differential cellular aging and found that distinct aging represent the proteomic signature of several diseases, including PD. Automation of this analysis using AI has shown promising potential for early detection of PD.

A summary of the techniques used for automated detection and staging of AD and PD is shown in Table 1.

**Table 1:** Summary of retinal imaging modalities and artificial intelligence (AI) approaches applied to alzheimer disease (AD) and Parkinson disease (PD)

Disease	Imaging modality	AI approach / model type	Main findings	Performance (as reported)	Strengths	Limitations
Alzheimer disease (AD)	Color fundus photography (CFP)	Vessel-based ML (SVM); DL (EfficientNet)	Microvascular alterations; small-vessel signatures; detection of MCI and AD	Tian et al.: 82% accuracy; Cheung et al.: ~80–92% accuracy	Non-invasive, accessible, captures vascular changes	Variable specificity; sensitive to image quality; systemic confounders
	OCT (structural)	XGBoost; feature-based ML	Decrease in RNFL, GCL, IPL; correlation with CSF biomarkers	~ 74% accuracy	High anatomical resolution; quantifiable biomarkers	Inter-device variability; segmentation dependence
	OCTA / Multimodal imaging	CNN multimodal	GC-IPL highly informative; perfusion changes	Wisely et al.: AUC ~ 0.83	Captures neurovascular and structural data	Requires high-quality multimodal imaging
Parkinson disease (PD)	Color fundus photography (CFP)	DL models (e.g., AlexNet)	Foveal and RNFL alterations; vessel-based signatures	~ 60–65% accuracy; low specificity	Widely available; captures early microvascular signals	Modest performance; high rate of false positives
	OCT / En-face texture	RF / texture-based ML	Textural changes in layers; superior macular texture alterations	~ 64–70% accuracy (across studies)	Sensitive to subtle microstructural features	Sensitive to artefacts and segmentation variability
	Multimodal (CFP, OCT, OCTA, UWF)	CNN multimodal	UWF color most informative; combining modalities improves accuracy	Richardson et al.: Sensitivity 100%, specificity 85%	Strongest overall performance; captures disease heterogeneity	High infrastructural demand; limited generalisability

The table synthesizes the principal imaging techniques investigated in the literature, the corresponding AI methodologies, reported diagnostic performance, and key strengths and limitations. The overview highlights the heterogeneity of existing approaches and the potential of multimodal imaging to improve early detection of neurodegenerative diseases

## Explainability techniques in retinal AI for neurodegenerative diseases

Explainability techniques are essential to ensure that automated classification of neurodegenerative diseases is biologically plausible and clinically trustworthy. Among them, Gradient-weighted Class Activation Mapping (Grad-CAM) and SHapley Additive exPlanations (SHAP) are the most widely used. Grad-CAM produces a localization heatmap by computing the gradients of the predicted class score with respect to the last convolutional layers, highlighting retinal regions that most strongly drive the model’s decision. In retinal oculomics, Grad-CAM has consistently revealed that AD classifiers focus on perimacular capillary networks, GC-IPL alterations, and small-caliber vessels. For example, Tian et al. demonstrated that attention maps derived from fundus imaging emphasized small vessels and capillaries, supporting the hypothesis that microvascular compromise reflects early AD-related neurovascular degeneration [19]. Similarly, Cheung et al. applied Grad-CAM to a DL fundus classifier for AD and confirmed that perifoveal arteriolar segments (not large vessels) were the most informative regions [22]. In multimodal imaging, Wisely et al. dissected network attention across OCT, OCTA, and UWF photographs, showing that the model consistently emphasized focal GC-IPL thinning patterns, in line with early RGC degeneration [26]. By contrast, SHAP provides feature-level interpretability by quantifying how each variable affects predictions. Wang et al. used SHAP to explain an XGBoost model trained on OCT B-scans and showed that TMV, GCL thickness, and IPL thickness were the most influential features, consistent with clinical correlations to CSF Aβ and p-tau levels [23]. In PD research, Nunes et al. applied SHAP to texture descriptors extracted from en-face OCT layers and found that superior macular texture disruptions were key discriminators between PD, AD, and controls. These patterns were not captured by conventional thickness metrics [24]. Together, Grad-CAM and SHAP provide complementary perspectives: the former clarifies where the model looks within retinal images, while the latter reveals which structural features drive classification. The fact that both align with known retinal signatures of AD and PD strengthens the biological credibility of AI-based retinal screening.

## Challenges, bias, and ethical considerations in retinal AI for neurodegeneration

Despite encouraging diagnostic performance, several structural and ethical barriers still limit the real-world deployment of retinal AI models for neurodegenerative diseases. Algorithmic bias is among the most critical issues. For instance, the UK Biobank (the dataset used in many influential AD and PD studies) has been repeatedly shown to be demographically non-representative, with over-representation of white, healthier, and socio-economically advantaged individuals [38]. As a consequence, AI models trained on Biobank fundus images, such as those by Tran et al. [32] for PD and Tian et al. [19] for AD, may exhibit reduced generalizability when applied to populations with different vascular phenotypes, ocular comorbidities, or imaging characteristics.

A second limitation concerns infrastructural and workflow constraints. Multimodal models, such as the ones proposed by Wisely et al. [26] and Richardson et al. [33], require high-quality OCT, OCTA, UWF imaging, and centralized data integration pipelines. These requirements challenge most non-tertiary hospitals, where OCTA adoption remains uneven and image quality control is rarely automated. Furthermore, the absence of harmonized acquisition protocols across centers creates variability that directly affects model reliability [39]. Moreover, inter-device variability remains a non-trivial challenge: recent cross-platform comparisons show that macular thickness and other quantitative OCT parameters differ systematically across instruments, with effect sizes large enough to limit direct interchangeability and to affect algorithmic performance trained on a single device type [40]. 

Finally, there are substantial ethical implications. Retinal AI may predict conditions, such as prodromal AD or PD, for which therapeutic pathways are still evolving. False positives, already observed in CFP-based PD detection [32], may trigger unnecessary neurological referrals, patient anxiety, and even downstream insurability or employment discrimination. Ethical guidance from the European Commission’s High-Level Expert Group on AI [41] emphasizes the need for transparency, human oversight, and strict governance when using AI in predictive health contexts. In the specific context of retinal OCT for neurology, Petzold et al. have proposed an extension of the OSCAR-IB quality criteria to AI applications (“OSCAR-AI”), explicitly warning that small, artefact-prone retinal changes demand rigorous quality control and governance before they can be used for automated neurodegenerative disease screening [42]. Addressing dataset bias, infrastructural inequality and these governance issues is therefore essential if retinal AI is to be deployed safely and equitably in clinical practice.

## Conclusions and perspectives

In consideration of the overall very good performance, AD and PD are likely to benefit from the support of retinal-imaging based AI for early diagnosis. To optimize the performance, especially in terms of specificity, the use of multimodal imaging and possible integration with meta-data has been demonstrated to be the most effective technique, particularly for PD diagnosis. However, in order to improve cost-effectiveness of a possible screening program, an ultimate selection of the most contributive imaging modalities is to be foreseen. Moreover, training and testing on a larger sample size and longitudinal evaluation of the chosen models will be needed before application on the general population. So far, explainability of the proposed models seems robust and coherent with the corresponding central nervous system damage. This correlation should remain under constant monitoring during the development of the final model, in order to avoid misdiagnosing an invalidating condition such as AD or PD due to spurious outcomes of the classifier [42]. 

In parallel, future research directions are expected to further refine the diagnostic value of retinal AI. The investigation of additional ocular changes predicting the onset of AD or PD may contribute to improving early diagnosis, including potential alterations in the vitreous or anterior segment, the detection of biomarkers visible with common imaging modalities, or the development of imaging techniques capable of assessing dopaminergic and cholinergic neuronal status within the retina. Beyond these avenues, the emergence of quantitative retinal biomarkers, such as microvascular indices from OCTA, widefield perfusion patterns, and layer-specific textural signatures, opens the possibility of identifying microstructural and microvascular alterations that precede overt anatomical loss. Similarly, advances in imaging technology, including widefield and swept-source OCT or hyperspectral devices, may allow detection of subtle biochemical shifts associated with early neurodegenerative pathology. Finally, the incorporation of longitudinal retinal trajectories and the integration of retinal AI outputs with systemic biomarkers, genetic information or cognitive data may ultimately support multimodal risk-stratification tools with improved specificity and clinical relevance.

## Data Availability

No datasets were generated or analysed during the current study.

## Abbreviations

AD: Alzheimer disease
CFP: Color Fundus Photograph
DL: Deep learning
FL: Feature learning
GC-IPL: Ganglion Cell Inner Plexiform Layer
INFD: Explanation infidelity
OCT: Optical Coherence Tomography
OCTA: Optical coherence tomography angiography
OPL: Outer Plexiform Layer
PD: Parkinson disease
ppRNFL: Peripapillary retinal nerve fiber layer
RGCs: Retinal ganglion cells
RPE: Retinal pigment epithelium
SCP: Superficial Capillary Plexus
SENS: Explanation sensitivity
SVM: Support Vector Machine
UWF: Ultrawide Field
